# Effect of blood flow restriction training in early postoperative rehabilitation after ACL reconstruction: a randomised controlled trial

**DOI:** 10.3389/fspor.2025.1689257

**Published:** 2026-01-14

**Authors:** Philipp Barzyk, Carolin Fiedler, Markus Schlag, Albrecht Heitner, Jonas Bender, Jochen Paul

**Affiliations:** Rennbahnklinik, Muttenz, Switzerland

**Keywords:** ACL, BFR, blood flow restriction, low-load training, rehabilitation

## Abstract

**Introduction:**

Anterior cruciate ligament (ACL) injuries represent the most prevalent type of knee injury, with surgical reconstruction being the prevailing treatment modality. However, postoperative pain and muscle weakness are common occurrences. Blood flow restriction (BFR) training has demonstrated potential in enhancing muscle adaptation and reducing pain; nevertheless, its efficacy in the early postoperative period following ACL reconstruction remains to be determined. The present study investigates whether the incorporation of BFR into low-load strength training confers additional clinical benefits in comparison to low-load exercise alone.

**Methods:**

A total of 30 patients (24 male, 6 female) with a mean age of 32.3 (± 12.4) years were included in the study. All patients had undergone primary ACL reconstruction with a semitendinosus graft and standardized fixation techniques. They were randomised to receive either low-load strength training with (LL-BFR) or without BFR (LL). The interventions consisted of four sets of leg press exercises (30 repetitions for the first set, 15 for subsequent sets) performed twice a week for four weeks, starting four weeks postoperatively, as an adjunct to standard rehabilitation protocols. The primary outcome measure was pain perception, which was assessed by pressure pain thresholds (PPT) and a visual analogue scale (VAS) for knee pain at rest and during a functional stair-climbing test. We further included strength and functional measurements.

**Results:**

Mixed linear models were employed for the statistical analysis. No statistically significant differences between groups were observed for the primary or secondary outcome measures. Only, significant time effects were found for both groups for subjective pain (rest *p* < 0.001, stairs *p* = 0.003); maximum strength (*p* = 0.002); active (ext. *p* = 0.035, flex. *p* < 0.001) and passive range of motion (ROM) (ext. *p* = 0.029, flex. *p* < 0.001) on the affected side and International Knee Documentation Committee (IDKC) scores (*p* < 0.001).

**Discussion:**

The present study shows that a combination of BFR and low-load strength training does not provide additional clinical benefits to low-load training alone in the early postoperative phase, following ACL reconstruction in our study group. Further research is required to explore the potential efficacy of BFR in specific patient subpopulations, with different training loads or at later stages of rehabilitation.

## 1 Introduction

With an incidence of more than 2 million injuries per year worldwide, anterior cruciate ligament (ACL) injuries are among the most common knee injuries (1, 2). In cases of functional impairment of the knee, reconstruction of the ACL is the current gold standard (3). The current evidence indicates that pain perception plays an important role in the development of postoperative knee function and a successful return to sport (4). Therefore, knee pain should be minimized in the early stages of rehabilitation, but optimal and generally accepted strategies and procedures for the control of perioperative pain are not well established (5).

In addition to the use of pain-reducing medication, studies have shown that exercise interventions can induce clinically relevant pain reduction (6), with a single training session being able to decrease pain sensations (7, 8). Studies in healthy individuals have shown that even low intensities are sufficient for reducing pain if strength training is paired with a temporary restriction of venous blood flow (9–14). This is also important when considering patient populations. While high-load training is often feasible for healthy, pain-free subjects, such high loads are contraindicated for ACL patients, particularly for the musculoskeletal system during the initial weeks following surgery.

Blood flow restriction (BFR) training is a training modality that is being used more and more frequently especially in rehabilitation of musculoskeletal injuries. The underlying principle is based on blocking venous return flow during strength training (15). The resulting occlusion can be induced, for example, using standard blood pressure cuffs or other cuff systems, which are attached to the proximal end of the limb to be trained. The occlusion pressure should be selected so that the venous return flow is prevented but the arterial inflow is maintained (16). This induces a local hypoxic environment without causing irreversible muscle damage (17, 18). Some studies argue, that training at low intensities (20%–30% of maximum strength) with additional restricted blood flow, can lead to comparable muscle cross-sectional adaptations and strength gains as conventional hypertrophy training (>70% of 1RM) without restriction (19–21). This is attributed to the fact that the low oxygen levels generated during this training increase the recruitment of glycolytic type II fibers, which have a high hypertrophy potential (22, 23) and are responsible for fast-twitch movements/contractions.

These positive effects on muscle hypertrophy and strength have been demonstrated in healthy individuals as well as in patients with pathologies of the knee joint (24, 25). In patient populations after ACL reconstruction, several studies consistently demonstrated that BFR training significantly increases muscle strength compared to similar non-BFR training or even high-load training (26–29). However, other research has reported mixed results or no significant differences in strength outcomes between BFR and control groups (25, 30–33). Some studies found significant increase or significantly less decrease in muscle mass with BFR training compared to non-BFR control groups (26, 27, 34, 35), whereas others found no differences between groups (25, 36). Studies that assessed functional outcomes and quality of life generally report improvement in both the BFR and control groups, without differences between them (26, 29–32). Regarding pain levels, several studies reported significant reductions in pain in BFR groups compared to high-load training, but not compared to low-load training alone (25, 26, 29, 37, 38). Other studies often found improvements within groups, with no significant differences in pain between BFR and control groups (28, 30, 32, 33, 39, 40).

Overall, there is some evidence to suggest that low-load BFR training could reduce pain and increase strength and knee function in patients with knee pathologies. Therefore, this study examined the effects of low-load strength training with (BFR-LL) against low-load training without (LL) BFR on pain perception during the early rehabilitation phase after ACL reconstruction. The primary objective of this study was to determine whether postoperative low-load training with BFR results in a more significant reduction in pain perception than low-load training without BFR. A secondary aim was to examine whether postoperative low-load training with BFR leads to a greater improvement in muscle function and structure than low-load training without BFR after surgery.

## 2 Materials and methods

### 2.1 Patients and selection criteria

**Figure 1:**
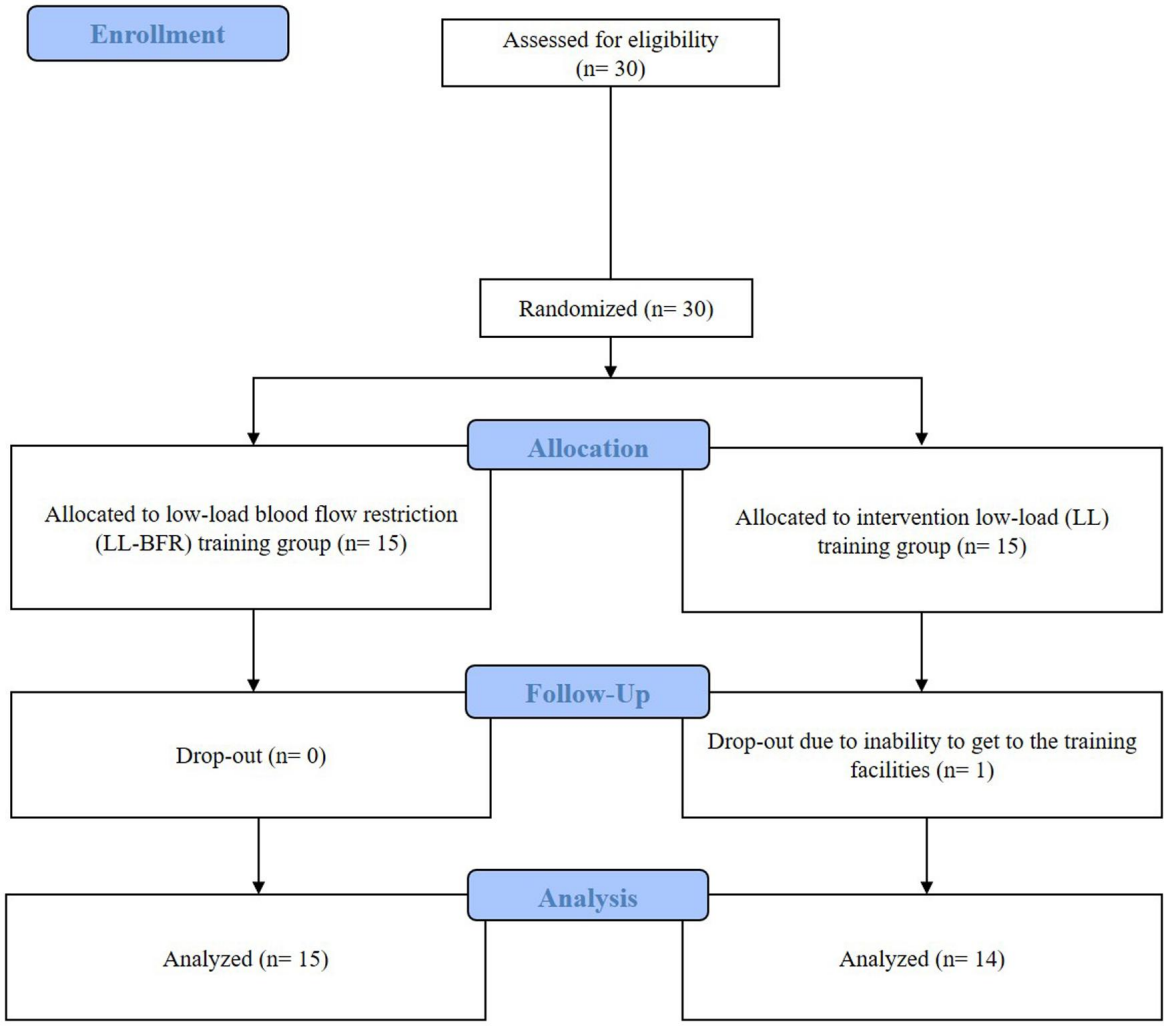
CONSORT flow diagram showing inclusion, randomization and participation throughout the study. Adapted from Hopewell et al. (463).

*N* = 30 patients with ACL reconstruction were recruited for this monocentric, parallel-group, randomised controlled trial with two arms and repeated pre- and post-measurements at the Rennbahnklinik in Muttenz, Switzerland. The required sample size (*N* = 30) was determined *a priori* for the primary outcome measure, for a mixed ANOVA, as calculated using G*Power Version 3.1. This was based on an effect size of d = 0.6 for changes in acute pain perception following BFR training, as reported in a previous study (11). This calculation indicated that *N* = 24 would provide 80% power (*α* < 0.05). To account for an anticipated 15%–20% dropout rate, 30 patients were finally recruited. Ultimately, only one patient withdrew from the trial due to an inability to attend the training at Rennbahnklinik (see Figure 1). All surgeries were performed exclusively by three senior surgeons using the same standard ACL reconstruction technique (semitendinosus graft and femoral-extracortical and tibial aperture screw fixation in combination with extracortical fixation).

The inclusion criteria for participation in this study included the following: patients must have undergone primary surgical anterior cruciate ligament (ACL) reconstruction; be aged between 18 and 60 years; have signed a written informed consent form; be able to stand on one leg unsupported for more than five seconds; have a knee range of motion between 0 and 90 degrees; and be able to perform a straight leg raise with sufficient quadriceps muscle activation. Exclusion criteria included chronic ACL rupture (more than six months); other ligamentous injuries requiring surgery; total meniscectomy; meniscal suturing or transplantation; surgically addressed cartilage injury; a history of thrombosis or current thrombosis; a prior infection and hypertension not controlled by medication.

The study was approved by the local ethics committee (2023–00151) and conducted in accordance with the Declaration of Helsinki. All testing data were encoded to ensure pseudonymization of the patients. The study was registered at ClinicalTrials.gov (ID: NCT06699264).

### 2.2 Study design

All examinations for this randomised controlled trial took place at Rennbahnklinik. Pre-testing was performed four weeks post-operatively, immediately before the first training session. Post-testing was performed within 2 days and one week after the last training session. Before the start of the intervention, the patients were randomised in blocks into two groups using a computer-generated sequence: low-load strength training with BFR (LL-BFR) or low-load strength training without BFR (LL). For both groups, training was performed twice a week in addition to the regular standardized rehabilitation program at the clinic (see Supplementary Material). A training session consisted of four sets of exercises on a leg press. The first set was performed with 30 repetitions and the following three sets with 15 repetitions. There was a 60-second rest between each set. While the first two weeks of training were performed at 20% of individual dynamic maximum strength, the intensity was increased to 30% of maximum strength in the last two weeks of training to ensure a progressive increase in load (41). To determine the training load, a 10-repetition maximum (10RM) test was carried out three times on a leg press machine after a standardized 5-minute warm-up on the cycle ergometer. The participants performed bilateral leg-press squats with a load that allowed for the completion of exactly ten controlled repetitions. The resulting 10RM value was then used to estimate the 1RM (as a 1RM measurement 4 weeks after surgery may represents a too intense strain), which subsequently served as the basis for calculating the training weight and the value of the dynamic maximum strength.

In the LL-BFR group, a pressure cuff (Tourniquet Cuff, VBM, Sulz, Deutschland) was additionally attached to the proximal end of the muscles to block venous return flow during training (11, 25). The cuff pressure was applied constantly for 8–10 min at 80% of the individual arterial occlusion pressure (AOP) using a tourniquet system during the entire exercise and rest period. The individual AOP was determined before every training session using Doppler ultrasound (Taschendoppler Handydop, Kranzbühler Medical Systems, Germany) on the posterior-tibial artery. Each training session was supervised by an experienced sports scientist. To ensure optimal recovery, two subsequent sessions were separated by a break of at least 48 h.

### 2.3 Measurement protocol

The primary outcome was pain perception, as measured by pressure pain threshold (PPT) and the visual analogue scale (VAS). For this purpose, an algometer (Wagner FPX Algometer, Wagner Instruments, Greenwich, United States of America) was used to examine the sensation of pain at four standardized localizations (dominant and non-dominant quadriceps muscle, dominant biceps brachii muscle, non-dominant trapezius muscle). Previous studies reported high reliability of a pressure algometer for assessing pain sensitivity (42–45). The investigator induced uniform pressure with a stimulation area of 1 cm^2^ and a pressure rate of 1 kgf/s. As soon as the patients felt the first pain perception, they were asked to report this verbally, whereupon the pressure was stopped. Two tests were carried out at each measurement point with an interval of 20 s. The average of the two values was used for the evaluation. As a subjective measure of knee joint pain, the perceived pain in the knee joint was further assessed using a VAS. The patients were asked to remember the most intense pain they experienced in their knee joint over the last week and rate it on the VAS. In order to evaluate a functional transfer of pain reduction, pain during a functional task (climbing stairs) was also assessed using VAS before and after the 4-week training intervention. Secondary outcomes included: Knee joint mobility determined using the neutral-zero method (active and passive flexion and extension), muscle thickness recorded using B-mode ultrasound (10 MHz, 5 mm transducer width) on the rectus femoris, dynamic maximum leg-press strength (10RM) and knee joint effusion (using joint circumference). Furthermore, knee functionality was recorded using the IKDC questionnaire, which evaluates patients' subjective knee condition by collecting information on symptoms, daily functional abilities, and sports activities (46).

### 2.4 Statistical analysis

Multivariate testing was used to address the research questions, with statistical significance determined at a *p*-value of less than 0.05. In cases of significance, adjusted *post-hoc* tests were conducted to further examine the differences. Descriptive statistics, including means and standard deviations, are reported for each group (LL-BFR and LL). Additionally, the F-statistic, *p*-value, and eta-squared (*η*^2^) are presented to indicate the results for the main effects of group, time, and their interaction [Group(x)Time]. An eta-squared (*η*^2^) > 0.01 is considered a small effect, > 0.06 a medium effect and >0.14 a large effect (47).

## 3 Results

**Figure 2:**
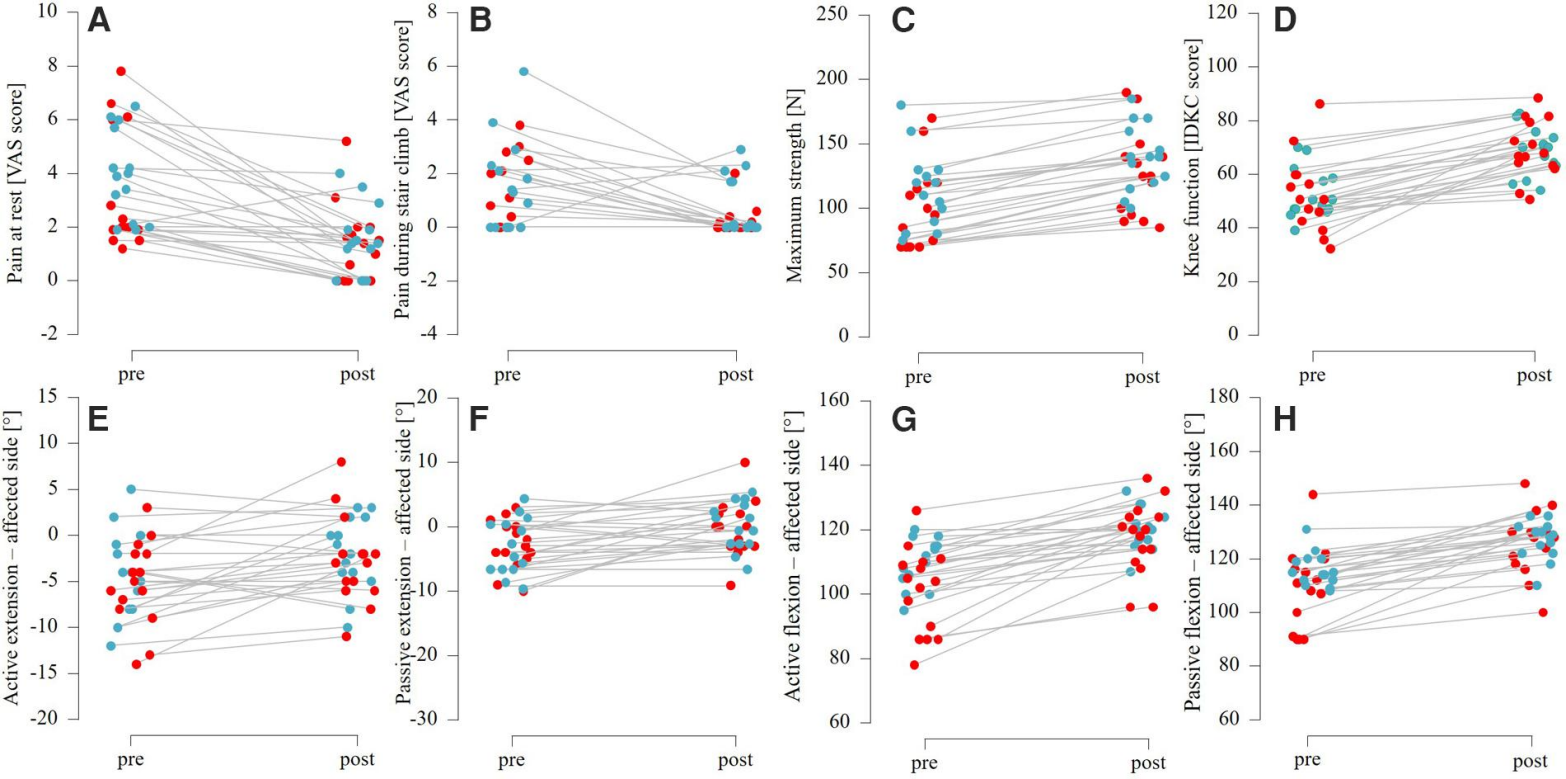
Pre-post differences between low-load blood flow restriction (in red) and low-load (in blue) training for: **A-B**) pain measurements; **C**) maximum strength in a leg-press; **D**) functional knee scoring; **E-H**) range of motion testing.

*N* = 29 patients (6 female, 23 male, aged 32.2 years ± 12.5 years) were included in the analysis. Recruitment began on 2 March 2023, and the first training session took place on 11 April 2023. The last participant completed their final training session on 16 December 2024, and the final study visit took place on 23 December 2024. Patient characteristics can be found in Table 1. Both LL-BFR and LL interventions resulted in significant improvements (see Table 2) in subjective pain, muscle strength, functional outcomes and range of motion (affected side). However, there were no significant differences between groups. Figures 2A–H demonstrates the difference in pre- and post-values for the above-mentioned parameters divided by group (LL and LL-BFR). In accordance with the pre-specified statistical analysis plan, only the planned primary and secondary analyses of the effects of group, time and the interaction between group and time were performed. No additional subgroup or sensitivity analyses were conducted because the available sample size was insufficient to permit adequately powered stratified or exploratory *post hoc* models. Further unplanned analyses were considered to be at high risk of yielding unstable or spurious findings.

**Table 1:** Patient characteristics divided by training group.

Descriptives	Mean & SD	
	LL-BFR	LL
Sex	m = 11, f = 4	m = 13, f = 2
Age [y]	34.7 ± 12.5	29.9 ± 12.4
Weight [kg]	75.5 ± 10.8	87.6 ± 13.2
Height [cm]	175.2 ± 6.8	180.2 ± 10.4
BMI [kg/m^2^]	24.61 ± 3.67	27.01 ± 3.95
Affected side	r = 11, l = 4	r = 8, l = 7

These include: sex, age in years, weight in kg, height in cm, Body-Mass-Index (BMI) in kg/m^2^ and the affected limb (left/right). Data is given as mean ± SD.

**Table 2:** Significant (*p* < 0.05) time effects for low-load blood flow restriction (LL-BFR) training and low-load (LL) training.

Group	Measurement	Pre	Post	F	*p*	*η* ^2^
LL-BFR:	Pain at rest [VAS score]	3.2 ± 2.2	1.3 ± 1.4	19.341	<.001	.258
LL:		3.7 ± 1.9	1.5 ± 1.3			
LL-BFR:	Pain during stair climb [VAS score]	2.4 ± 2.8	0.2 ± 0.5	9.789	.003	.145
LL:		1.5 ± 1.7	0.8 ± 1.1			
LL-BFR:	Maximum strength [N]	100.3 ± 32.4	126.3 ± 32.3	10.620	.002	.155
LL:		114.3 ± 29.1	139.3 ± 25.3			
LL-BFR:	Knee function [IDKC score]	51.2 ± 14.7	69.2 ± 10.9	29.456	<.001	.351
LL:		53.9 ± 10.2	68.4 ± 8.8			
LL-BFR:	Active extension – affected side [ °]	−5.2 ± 4.6	−2.7 ± 4.7	4.693	.035	.078
LL:		−4.6 ± 4.8	−1.9 ± 4.0			
LL-BFR:	Passive extension – affected side [ °]	−2.8 ± 3.8	−0.4 ± 4.4	7.041	.010	.112
LL:		−2.4 ± 4.3	0.8 ± 3.7			
LL-BFR:	Active flexion – affected side [ °]	104.9 ± 13.3	120.0 ± 11.7	22.627	<.001	.284
LL:		110.1 ± 7.6	120.4 ± 6.4			

Values are presented as mean plus standard deviation results. Effect size for the ANOVA statistics is presented as eta-squared (η^2^ > 0.01 is considered a small effect, η^2^ > 0.06 a medium effect and η^2^ > 0.14 a large effect).

## 4 Discussion

This study examined the effects of low-load blood flow restriction (LL-BFR) training vs. low-load (LL) training without BFR in the early postoperative phase following ACL reconstruction. The findings of the present study demonstrate that both interventions resulted in significant improvements over time in subjective pain, muscle strength, range of motion (ROM), and knee function.

The primary question was whether low-load training with BFR leads to a significant reduction in pain perception compared to low-load training without BFR. However, the objective [i.e., the pressure pain thresholds (PPT) measured with an algometer] and subjective (i.e., VAS) pain measures did not demonstrate significant group differences. The observed outcomes may be attributable to the initial low pain ratings on the VAS. The VAS scores for assessing pain in daily life were 3.2 ± 2.2 in the intervention group and 3.7 ± 1.9 in the control group. A similar picture emerges for pain experienced during the functional task. While patients in the intervention group reported VAS scores of 2.4 ± 2.8 at baseline, patients in the control group reported scores of 1.5 ± 1.7. Consequently, the pain perception levels in the control group were found to be minimal at baseline, a factor that should be considered when interpreting the results.

In addition, the assessment of pain perception using PPTs for patients with anterior cruciate ligament (ACL) injuries might not be appropriate. Previous studies which showed significant results for a reduced (acute) pain perception in the knee joint after BFR training have assessed pain using only a pain scale (30, 38, 48, 49). Furthermore, Lau et al. (50) and Sanches et al. (51) who compared VAS and PPT, found no correlation between them and argue that both measures represent different aspects of pain (50, 51). More specifically, Sanches et al. (51) argue that VAS represents the intensity of pain, whereas PPT represents the sensitivity to pain or the threshold of minimal pain intensity (51). Therefore, subsequent studies should consider which aspect of pain to assess and therefore choose the correct subjective (VAS) or objective (PPT) measurement.

The overall findings in this interventional study are consistent with others which found improvements for BFR and control groups with no significant differences between them (28, 30, 32, 33, 39, 40). However, other studies have reported significant reductions in pain in BFR groups compared to control, though not necessarily compared to low-load training alone (25, 26, 29, 37, 38).

Our results regarding increased muscle strength align with some current literature reporting mixed or inconclusive findings regarding strength outcomes between BFR and control groups (25, 30–33). However, they contrast with other studies that have demonstrated that BFR training significantly increases muscle strength compared to similar non-BFR training or even high-load training (26–29). Similarly, the improvements in knee function observed in both the LL-BFR and LL group are consistent with the broader literature who previously reported improvements without significant group differences (26, 29–32).

Recent publications have summarized the effects of blood flow restriction (BFR) training on anterior cruciate ligament (ACL) injuries and their recovery in various settings. However, in accordance with the information above, their results concerning pain, muscle strength, cross-sectional area (CSA), and functional outcomes vary considerably, which makes it challenging to develop recommendations for future study methodology (52–55). In particular, little information is available on the long-term effects on pain perception, as many studies solely report immediate effects of BFR training (sometimes after a single session) on pain sensitivity. Therefore, controlled trials, systematic reviews and meta-analyses on studies examining the (longitudinal) effects of BFR training on pain in both healthy and pathological populations are needed.

The lack of significant group differences we observed may also be explained by the relatively low training load (20% of 1RM, increased to 30% in the last two training weeks) and bilateral exercise protocol used in this study. In 2023, the German Federal Institute of Sports Science (BISp) published a position paper on blood flow restriction (BFR) training, recommending mechanical resistance intensities of 20%–50% of an individual's 1RM (56). They based their recommendation on two studies; however, their results are open to interpretation. The first study found that, while low-load training (15% 1RM), with or without BFR, stimulated muscle growth, high-load training (70% 1RM) produced more substantial gains. Importantly, only high-load training increased muscle strength (1RM), suggesting that BFR alone is insufficient to improve strength (57). The second study found that adding BFR to high- or moderate-intensity strength training did not enhance muscle hypertrophy or strength gains compared to training without BFR. The authors suggest that the muscle tension and metabolic stress from high-load training (>50% 1RM) are sufficient to maximize these adaptations, rendering additional occlusion unnecessary at higher loads. This study only included eight participants per group, who performed sixteen training sessions in total. The authors also questioned the transferability of their results to bilateral movements (58). Together, these results seem insufficient to recommend BFR training at intensities of 15%–50% 1RM, highlighting the uncertainty in the current literature.

It should also be acknowledged that the absence of significant group differences may reflect limited statistical power rather than a true lack of effect. Given the relatively small sample size and variability in individual pain responses, the study was primarily designed to detect significant effects. Consequently, smaller, yet potentially meaningful, differences between groups may have gone undetected.

Our study has several additional limitations that should be considered, like an uneven gender distribution, with only six females. This could have influenced pain ratings, given known gender differences in pain sensitivity (59). It should also be noted that the BMI is higher in the control group. The significantly lower body weight of the intervention group could have influenced the results, and this should be accounted for in future studies. Blinding of patients and assessors was not feasible, potentially introducing expectation bias. Additionally, low baseline pain in both groups (see Figures 2ii-iii) may have led to a floor effect, limiting the potential for further reductions and complicating interpretation. In addition, the training intensity appears to have been too low. This may be because our methodology was partly based on an RCT (41), which referred to four studies examining various types of training in older people (19, 60–62). However, only Karabulut et al. (19) and Yasuda et al. (62) examined BFR and their results (an increase in muscle strength and mass) do not appear to be transferable to clinical populations (19, 62). Nonetheless, other studies also investigated the effects of BFR training with similar intensities and reported varying results. Further studies are therefore needed to determine which, training intensities are most effective for clinical populations.

### 4.1 Conclusions

This study examined the effects of low-load blood flow restriction (LL-BFR) training on pain perception, knee joint mobility, muscle strength, and functional performance in patients after anterior cruciate ligament (ACL) reconstruction surgery. Although significant improvements were observed over time, no differences were found between LL-BFR and low-load (LL) training. These results contradict some previous research indicating positive effects of BFR training on pain reduction and other rehabilitation parameters but align with other studies reporting varying results. The findings of this study may suggest that the training stimulus used, involving a relatively low load (20%–30% of 1RM) and bilateral exercises, may have been insufficient in postoperative ACL patients. This study does not yet provide clear evidence to support the use of LL-BFR training for patients in the early stages after ACL reconstruction. While BFR training may offer benefits, further large-scale, longitudinal research is needed to assess the impact of BFR training at different intensities and with different patient populations before recommendations can be made on its use. Future studies should address the limitations of this study by increasing the sample size, ensuring gender balance, implementing blinding where possible, and providing clear recommendations on methodology for specific populations.

## Data Availability

The raw data supporting the conclusions of this article will be made available by the authors, without undue reservation.
