# Supplementary material for: Effect of blood flow restriction training in early postoperative rehabilitation after ACL reconstruction: a randomised controlled trial
Source: Front Sports Act Living. 2026 Jan 14;7:1689257. doi: 10.3389/fspor.2025.1689257 (PMC12847322; doi:10.3389/fspor.2025.1689257)
Supplement: Supplementary file 1 [file Supplementaryfile1.pdf]

Rennbahnklinik

Praxisklinik Rennbahn AG
Kriegackerstrasse 100
CH-4132 Muttenz
Tel: +41 (0)61 465 64 64
Fax: +41 (0)61 465 64 65
info@rennbahnklinik.ch
www.rennbahnklinik.ch

Physiotherapie
Tel: +41 (0)61 465 64 44

swiss olympic MEDICAL CENTER

Nachbehandlungsschema: Rekonstruktion des vorderen Kreuzbandes (VKB)

*Die Evaluations- und Progressionskriterien werden jeweils am Ende der Rehabilitationsphase erhoben.
Werden die Evaluations- und Progressionskriterien ab Phase 2 nicht erfüllt, verzögert sich der gesamte Rehabilitationsverlauf.

Phase	Ziele nach ICF	Behandlungsmassnahmen	Evaluations- und Progressionskriterien*
**Präoperativ**		Schmerzmanagement<br>Abschwellende Massnahmen<br>Proprioceptive Übungen<br>Quadriceps-Training<br>Information über Rehabilitationsverlauf nach OP	Quadriceps-Index (Isokinetik[1])
Präoperative Visitentermine in der Rennbahnklinik: Anästhesie und Labor (ca. 60 min), Biomechanik (30min)			
**Phase 1**<br>(Woche 1 und 2)	**Struktur**<br>Reduktion der Schmerzen<br>Reduktion der Schwellung<br><br>**Funktion**<br>Beweglichkeit Flexion/Extension (90/0/0)<br>Isometrische Aktivierung Quadriceps<br>Belastungsaufbau<br><br>**Aktivität**<br>Steigerung der Alltagsaktivität	Schmerzmanagement<br>Abschwellende Massnahmen<br>Passive und aktive Kniemobilisation<br>Mobilisation der Patella<br>Isometrische Aktivierung m. quadriceps femoris<br>Hamstringsdehnung bei fehlender Extension (prone hangs<br>Gangschulung (3-Punkte Gang an Stöcken, inkl. Treppe)<br>Proprioceptive Übungen beidbeinig<br>Fahrradergometer (max. 70 Umdrehungen pro Minute)<br>Compex[3]	Ergusstest[2]<br>Entzündungszeichen<br>Beweglichkeit Flexion/Extension 90/0/0 passiv<br>Quadriceps-Isometrie mit Cranialgleiten der Patella<br>Volle Knieextension (auch beim Gehen)<br>Korrekte Streckhebung (SLR) ohne Extensionsverlust<br>• Sobald i.O Vollbelastung erlaubt
2 Wochen postoperativ Fadenentfernung in der Rennbahnklinik oder beim Hausarzt			
**Phase 2**<br>(Woche 3 bis 6)	**Funktion**<br>Beweglichkeit Flexion/Extension (>110/0/0)<br>Verbesserte Muskelkoordination<br><br>**Aktivität**<br>Normalisierung des Gangbildes<br>Alternierendes Treppensteigen	Aktive und passive Kniemobilisation<br>Narbenmobilisation<br>Gangschulung ohne Stöcke<br>Proprioceptive Übungen auch im Einbeinstand<br>Koordination in geschlossener Kette (Squat, Squat Lunges, ...)<br>Training Rumpf- und Hüftstabilisatoren<br>Fahrradergometer<br>Compex[3]	Ergusstest minim[2]<br>Knieflexion >110°<br>Normales Gangbild<br>Alternierendes Treppensteigen
6 Wochen postoperativ Visitentermine in der Rennbahnklinik: Arzt (30min)			
**Phase 3**<br>(ca.Woche 7-12)	**Funktion**<br>Seitengleiche Knie-Beweglichkeit<br><br>**Aktvität**<br>Fahrradfahren draussen<br>Crawl und Rückenschwimmen	Kraftaufbau (Maximalkraft) in geschlossener und offener Kette (Widerstände an proximaler Tibia setzen)<br>Intensivierung proprioceptiver Übungen<br>Lauf und Sprung-ABC mit stabiler Beinachse<br>Joggen (Beginn wenn Lauf-und Sprung-ABC beschwerdefrei)	Knieflexion innerhalb 10° der anderen Seite<br>Level 1 Test: Balance Squat, Y-Balance Test<br>Level 2 Test: Balance Front Hop
12 Wochen postoperativ Visitentermine in der Rennbahnklinik: Physiotherapie (30min), Arzt (30min)			
**Phase 4**<br>(ca. Woche 13 bis Ende 6. Monat)	**Funktion**<br>Schmerzfreier Dehnungsverkürzungszyklus<br><br>**Aktivität**<br>Joggen outdoor	Intensivierung Lauf und Sprung-ABC; Sprünge in der Frontalebene (Cutting Bewegungen), bei stabiler Bein-achse auch repetitierend bis zur Ermüdung<br>Weiterführung Maximalkraftaufbau<br>Schnellkraft	Quadriceps-Index (Isokinetik[1])<br>Level 2 Test: Balance Front Hop, Front Hop Test<br>Level 3 Test: Balance Side Hop, Side Hop Test
6 Monate postoperativ Visitentermine in der Rennbahnklinik: Physiotherapie (30min), Biomechanik (30min), Arzt (30min)			

▶▶

Phase	Ziele nach ICF	Behandlungsmassnahmen	Evaluations- und Progressionskriterien*
**Phase 5**<br>(7. Monat bis Ende 9. Monat)	**Aktivität**<br>Sportartspezifische Bewegungsabläufe<br><br>**Partizipation**<br>Return to sport	Multidirektionale konzentrische und exzentrische Sprungbelastungen, repetitierend bis zur Ermüdung<br>Dehnungsverkürzungszyklus, Plyometrie<br>Sportartspezifische Bewegungen<br>Stop and Go Belastungen<br>Kontinuierlicher, gecoachter Return to sport:<br>- sportartspezifische Übungen in gewohnter Trainingsumgebung<br>- alle Übungen ohne Körperkontakt	Quadriceps-Index (evtl. Isokinetik, Hop Tests[4])<br>Level 3 Test: Balance Side Hop<br>Level 4 Test: 90° Balance Hop, Square Hop Test
9 Monate postoperativ Visitentermine in der Rennbahnklinik: Physiotherapie (30min), Biomechanik (60min), Arzt (30min)			
**Phase 6**<br>(ab 10. Monat)	**Partizipation**<br>Return to play and competition	Return to play (Rückkehr zur vollen Sportfähigkeit):<br>uneingeschränkte Teilnahme am Mannschaftstraining<br>Übungen mit Körperkontakt, Pressschlag und Tackling<br>Aufbau ausreichender Fitness (Ausdauer, Kraft, etc.)<br><br>Return to competition:<br>langsamer Aufbau der Dauer oder dem gesamten Umfang am Wettkampf	
1 Jahr postoperativ Visitentermine in der Rennbahnklinik: Arzt (30min), Biomechanik und Physiotherapie nur auf ärztliche Verordnung			

1 Erhoben mit Cybex isokinetic machine®. Protokoll Isokinetik: konzentrisch-konzentrisch, 60°/sec

2 Modified Stroke Test (Ergusstest): Erguss wird eingeteilt in fünf Stufen (0, minim, +, ++, +++)

3 Neuromuskuläre Stimulation mit Compex®, Programm Muskelatrophie, 2x pro Tag im Langsitz, ab Woche 3 im Sitz mit hängendem Bein (siehe Beiblatt)

4 Zwei verschiedene einbeinige Sprünge und Drop Jump beidbeinig (Reid 2007, Thomeé 2012, Logerstedt 2012)

Literatur:

Adams, D., Logerstedt, D., Hunter-Giordano, A., Axe, M. J., & Snyder-Mackler, L. (July 2012). Current Concepts for Anterior Cruciate ligament Reconstruction: A Criterion-Based Rehabilitation Progression. journal of Orthopaedic & Sports Physical Therapy, S. 601-614..

Collins, N. J., Misra, D., Fellson , D. T., Corssely, K. M., & Roos, E. M. (2011). ... Arthritis Care & Research, S. 208-28.

Diemer, F., & Sutor, V. (2011). Praxis der medizinischen Trainingstherapie I: Lendenwirbelsäule, Sakroiliakalgelenk und untere Extremität.

Fukuda, T. Y., Fingerhut, D., Moreira, V. C., Ferreira Camarini, P. M., Folco Scodeller, N., Duarte, A., et al. (2. April 2013). Open Kinetic Chain Exercises in a Restricted Range of Motion After Anterior Cruciate Ligament Reconstruction: A Randomized Controlled Clinical Trial. The American Journal of Sports Medicine, S. 788-794..

Herbst, E., Hoser, C., Hildebrandt, C., Raschner, C., Hepperger, C., Pointer, H., et al. (2015). Functional assessments for decision-making regarding return to sports follwoing ACL reconstruction. Part II: clinical application of a new test battery. Knee Surgery Sports Traumatology Arthroscopy, 1283-1291.

Hildebrandt, C., Müller, L., Zisch, B., Huber, R., Fink, C., & Raschner, C. (2015). Functional assessments for decision-making regarding return to sports following ACL reconstruction. Part I: development of a new test battery. Knee Surgery Sports Traumatology Arthroscopy, 1273-1281.

Keller, M., & Kurz, E. (2016). Zurück zum Pre Injury Level nach Verletzungen der unteren Extremität - eine Einteilung funktioneller Assessments. Manuelle Therapie, 16-18.

Keller, M., Kurz, E., Schmidtlein, O., Welsch, G., & Anders, C. (2016). Interdisziplinäre Beurteilungskriterien für die Rehabilitation nach Verletzungen an der unteren Extremität: Ein funktionsbasierter Return-To-Activity Algorithmus. Physikalische Medizin Rehabilitationsmedizin Kurortmedizin, 137-148.

Kruse, L. M., Gray, B., & Wright, R. W. (2012). Rehabilitation After Anterior Cruciate Ligament Reconstruction. The Journal of Bone and Joint Surgery, S. 1737-1748.

Myer, G. D., Paterno, M. V., Ford, K. R., Quatman, C. E., & Hewett, T. E. (2006). Rehabilitation after anterior cruciate ligament reconstruction: criteria-based progression through the return to sport phase. Journal of Sports Physical Therapy, S. 385-402.

Noyes, F. R., Darber, S. D., & Mangine, R. E. (1991). Abnormal lower limb symmetry determined by function hop tests after anterior cruciate ligament rupture. The American Journal of Sports Medicin, S. 513-518.

Reid, A., Birmingham, T., Stratford, P., Alcock, G., & Giffin, J. (20. February 2007). Hop testing provides a reliable and valid outcome measure during rehabilitation after anterior cruciate ligament reconstruction. Physical Therapy, S. 337-49.

Sturgill, L. P., Snyder-Mackler, L., Manal, T. J., & Axe, M. J. (2009). Interrater reliability of a clinical scale to assess knee joint effuction. Journal of Orthopaedic Sports Physical Therapy, S. 513-518.

Thomeé, R., Kaplan, Y., Kvist, J., Myklebust, G., Risberg, M., Theisen, D., et al. (19. November 2011). Muscle strength and hop performance criteria prior to return to sports after ACL reconstruction. Knee Surgery, Sports Traumatology, Arthroscopy, S. 1798-805.

Thomeé, R., Neeter, C., Gustavsson, A., Thomeé, P., Augustsson, J., Erisksson, B., et al. (June 2012). Variability in leg musclepower and hop performance after anterior cruciate ligament reconstruction. Knee Surgery, Sports Traumatology, Arthroscopy, S. 1143-51.

Wright, R. W., Prestion, E., Fleming, B., Amendola, A., Andrish, J. T., Bergfeld , J. A., et al. (July 2008). ACL Reconstruction Rehabilitation: A Systematic Review Part II. Journal of Knee Surgery, S. 225-234.
