# Supplementary material for: Effect of blood flow restriction training in early postoperative rehabilitation after ACL reconstruction: a randomised controlled trial
Source: Front Sports Act Living. 2026 Jan 14;7:1689257. doi: 10.3389/fspor.2025.1689257 (PMC12847322; doi:10.3389/fspor.2025.1689257)
Supplement: Supplementary file 2 [file Supplementaryfile2.pdf]

Rennbahnklinik

Praxisklinik Rennbahn AG
Kriegackerstrasse 100
CH-4132 Muttenz
Tel: +41 (0)61 465 64 64
Fax:+41 (0)61 465 64 65
info@rennbahnklinik.ch
www.rennbahnklinik.ch

Physiotherapie
Tel: +41 (0)61 465 64 44

swiss olympic MEDICAL CENTER

Follow-up treatment plan: Anterior cruciate ligament (ACL) reconstruction

*The evaluation and progression criteria are assessed at the end of each rehabilitation phase.
If the evaluation and progression criteria are not met from phase 2 onwards, the entire rehabilitation process will be delayed.

Phase	Goals according to ICF	Treatment measures	Evaluation and progression criteria*
Preoperativ		Pain management<br>Decongestant measures<br>Proprioceptive exercises<br>Quadriceps training<br>Information about the rehabilitation process after surgery	Quadriceps index (isokinetics¹)
Preoperative appointments at Rennbahnklinik: Anaesthesia and laboratory (approx. 60 min), biomechanics (30 min)			
Phase 1 (Weeks 1 and 2)	Structure<br>Reduction of pain<br>Reduction of swelling<br><br>Function<br>Mobility flexion/extension (90/0/0)<br>Isometric activation<br>Quadriceps load build-up<br><br>Activity<br>Increase in everyday activity	Pain management<br>Measures to reduce swelling<br>Passive and active knee mobilisation<br>Mobilisation of the patella<br>Isometric activation of the quadriceps femoris<br>Hamstring stretching in the absence of extension<br>Gait training (3-point gait with sticks, including stairs)<br>Proprioceptive exercises with both legs<br>Exercise bike (max. 70 revolutions per minute)<br>Compex³	Effusion test²<br>Signs of inflammation<br>Mobility Flexion/extension 90/0/0 passive<br>Quadriceps-isometry with cranial gliding of the patella<br>Full knee extension (also when walking)<br>Correct straight leg raise (SLR) without loss of extension<br>-> Once OK, full weight-bearing allowed
2 weeks post-op Suture removal at Rennbahnklinik or by your GP			
Phase 2 (Week 3 to 6)	Function<br>Mobility Flexion/extension (>110/0/0)<br>Improved muscle coordination<br><br>Activity<br>Normalisation of gait pattern<br>Alternating stair climbing	Active and passive knee mobilisation<br>Scar mobilisation<br>Gait training without crutches<br>Proprioceptive exercises, including standing on one leg<br>Closed chain coordination (squats, squat lunges, etc.)<br>Training of core and hip stabilisers<br>Exercise bike<br>Compex³	Effusion test minim²<br>Knee flexion >110<br>Normal gait pattern<br>Alternating stair climbing
6 weeks post-op appointment at Rennbahnklinik: Doctor (30 min)			
Phase 3 (approx. week 7-12)	Function<br>Knee mobility on both sides<br><br>Activity<br>Cycling outdoors<br>Crawl and backstroke swimming	Strength training (maximum strength) in closed and open chains (set resistance on proximal tibia)<br>Intensification of proprioceptive exercises<br>Running and jumping exercises with stable leg axis<br>Jogging (start when running and jumping exercises are pain-free)	Knee flexion within 10° of the other side<br>Level 1 test: balance squat, Y-balance test<br>Level 2 test: balance front hop
12 weeks post-op appointment at Rennbahnklinik: Physiotherapy (30 min), doctor (30 min)			
Phase 4 (approx. week 13 to end of 6th month)	Function<br>Pain-free stretch and shortening cycle<br><br>Activity<br>Outdoor jogging	Intensification of running and jumping basics; jumps in the frontal plane (cutting movements), with stable leg axis, also repetitive until fatigue<br><br>Continuation of maximum strength training<br>Speed strength	Quadriceps-Index (Isokinetik¹)<br>Level 2 Test: Balance Front Hop, Front Hop Test<br>Level 3 Test: Balance Side Hop, Side Hop Test

►►

Phase	Goals according to ICF	Treatment measures	Evaluation and progression criteria*
6 months post-op appointment at Rennbahnklinik: Physiotherapy (30 min), biomechanics (30 min), doctor (30 min)			
Phase 5<br>(7th month to end of 9th month)	Activity<br>Sport-specific movement sequences<br><br>Participation<br>Return to sport	Multidirectional concentric and eccentric jumping loads, repeated until fatigue<br>Stretch-shortening cycle, plyometrics<br>Sport-specific movements<br>Stop and go loads<br>Continuous, coached return to sport:<br>- sport-specific exercises in a familiar training environment<br>- All exercises without physical contact	Quadriceps index (possibly isokinetics, hop tests[4])<br>Level 3 test: balance side hop<br>Level 4 test: 90° balance hop, square hop test
9 months post-op appointment at Rennbahnklinik: Physiotherapy (30 min), biomechanics (60 min), doctor (30 min)			
Phase 6<br>(from 10th month)	Participation<br>Return to play and competition	Return to play (return to full sporting ability):<br>unrestricted participation in team training exercises involving physical contact, pressing and tackling<br>Building up sufficient fitness (endurance, strength, etc.)<br>Return to competition:<br>Slow build-up of duration or total volume in competition	
1 year post-op appointment at Rennbahnklinik: Doctor (30 min), biomechanics and physiotherapy only on medical prescription			

1 Measured with Cybex isokinetic machine®. Isokinetic protocol: concentric-concentric, 60°/sec

2 Modified stroke test (effusion test): effusion is divided into five stages (0, minim, +, ++, +++)

3 Neuromuscular stimulation with Compex®, muscle atrophy programme, twice a day in a long sitting position, from week 3 in a sitting position with legs hanging down

4 Two different single-leg jumps and drop jumps with both legs (Reid 2007, Thomeé 2012, Logerstedt 2012)

Literature:

Adams, D., Logerstedt, D., Hunter-Giordano, A., Axe, M. J., & Snyder-Mackler, L. (July 2012). Current Concepts for Anterior Cruciate ligament Reconstruction: A Criterion-Based Rehabilitation Progression. Journal of Orthopaedic & Sports Physical Therapy, S. 601-614..

Collins, N. J., Misra, D., Fellson , D. T., Corssely, K. M., & Roos, E. M. (2011). ... Arthritis Care & Research, S. 208-28.

Diemer, F., & Sutor, V. (2011). Praxis der medizinischen Trainingstherapie I: Lendenwirbelsäule, Sakroiliakalgelenk und untere Extremität.

Fukuda, T. Y., Fingerhut, D., Moreira, V. C., Ferreira Camarini, P. M., Folco Scodeller, N., Duarte, A., et al. (2. April 2013). Open Kinetic Chain Exercises in a Restricted Range of Motion After Anterior Cruciate Ligament Reconstruction: A Randomized Controlled Clinical Trial. The American Journal of Sports Medicine, S. 788-794..

Herbst, E., Hoser, C., Hildebrandt, C., Raschner, C., Hepperger, C., Pointer, H., et al. (2015). Functional assessments for decision-making regarding return to sports follwoing ACL reconstruction. Part II: clinical application of a new test battery. Knee Surgery Sports Traumatology Arthroscopy, 1283-1291.

Hildebrandt, C., Müller, L., Zisch, B., Huber, R., Fink, C., & Raschner, C. (2015). Functional assessments for decision-making regarding return to sports following ACL reconstruction. Part I: development of a new test battery. Knee Surgery Sports Traumatology Arthroscopy, 1273-1281.

Keller, M., & Kurz, E. (2016). Zurück zum Pre Injury Level nach Verletzungen der unteren Extremität - eine Einteilung funktioneller Assessments. Manuelle Therapie, 16-18.

Keller, M., Kurz, E., Schmidtlein, O., Welsch, G., & Anders, C. (2016). Interdisziplinäre Beurteilungskriterien für die Rehabilitation nach Verletzungen an der unteren Extremität: Ein funktionsbasierter Return-To-Activity Algorithmus. Physikalische Medizin Rehabilitationsmedizin Kurortmedizin, 137-148.

Kruse, L. M., Gray, B., & Wright, R. W. (2012). Rehabilitation After Anterior Cruciate Ligament Reconstruction. The Journal of Bone and Joint Surgery, S. 1737-1748.

Myer, G. D., Paterno, M. V., Ford, K. R., Quatman, C. E., & Hewett, T. E. (2006). Rehabilitation after anterior cruciate ligament reconstruction: criteria-based progression through the return to sport phase. Journal of Sports Physical Therapy, S. 385-402.

Noyes, F. R., Darber, S. D., & Mangine, R. E. (1991). Abnormal lower limb symmetry determined by function hop tests after anterior cruciate ligament rupture. The American Journal of Sports Medicin, S. 513-518.

Reid, A., Birmingham, T., Stratford, P., Alcock, G., & Giffin, J. (20. February 2007). Hop testing provides a reliable and valid outcome measure during rehabilitation after anterior cruciate ligament reconstruction. Physical Therapy, S. 337-49.

Sturgill, L. P., Snyder-Mackler, L., Manal, T. J., & Axe, M. J. (2009). Interrater reliability of a clinical scale to assess knee joint effuction. Journal of Orthopaedic Sports Physical Therapy, S. 513-518.

Thomeé, R., Kaplan, Y., Kvist, J., Myklebust, G., Risberg, M., Theisen, D., et al. (19. November 2011). Muscle strength and hop performance criteria prior to return to sports after ACL reconstruction. Knee Surgery, Sports Traumatology, Arthroscopy, S. 1798-805.

Thomeé, R., Neeter, C., Gustavsson, A., Thomeé, P., Augustsson, J., Erisksson, B., et al. (June 2012). Variability in leg musclepower and hop performance after anterior cruciate ligament reconstruction. Knee Surgery, Sports Traumatology, Arthroscopy, S. 1143-51.

Wright, R. W., Prestion, E., Fleming, B., Amendola, A., Andrish, J. T., Bergfeld , J. A., et al. (July 2008). ACL Reconstruction Rehabilitation: A Systematic Review Part II. Journal of Knee Surgery, S. 225-234.
